# The Association between Altitude and Waist–Height Ratio in Peruvian Adults: A Cross-Sectional Data Analysis of a Population-Based Survey

**DOI:** 10.3390/ijerph191811494

**Published:** 2022-09-13

**Authors:** Akram Hernández-Vásquez, Diego Azañedo

**Affiliations:** 1 Centro de Excelencia en Investigaciones Económicas y Sociales en Salud, Vicerrectorado de Investigación, Universidad San Ignacio de Loyola, Lima 15024, Peru; 2 Facultad de Ciencias de la Salud, Universidad Científica del Sur, Lima 15067, Peru

**Keywords:** weight–height ratio, altitude, cardiometabolic risk factors, cross-sectional studies, Peru

## Abstract

To evaluate the association between altitude and cardiometabolic risk calculated with the weight–height ratio (WHtR) in the Peruvian adult population via the cross-sectional data analysis of the Peruvian Demographic and Health Survey 2021. A total of 26,117 adults from 18 to 64 years of age were included in the analysis. The dependent variable was cardiometabolic risk, defined as “Yes” if the WHtR was ≥0.5 and “No” if the WHtR was <0.5. Exposure was altitude of residence categorized as: <1500 meters above sea level (masl); 1500 to 2499 masl; 2500 to 3499 masl; and ≥3500 masl. Crude and adjusted Poisson regression models were used to calculate prevalence ratios (PR) with 95% confidence intervals (CI). The mean WHtR in the population was 0.59 (standard deviation: 0.08), and 87.6% (95% CI: 86.9–88.2) were classified as at risk. After adjusting for sex, age, education level, well-being index, and area of residence, living at altitudes between 2500 and 3499 masl (aPR: 0.98; 95% CI: 0.96–1.00) and ≥3500 masl (aPR: 0.95; 95% CI: 0.93–0.97) were associated with lower cardiometabolic risk in comparison with living at <1500 masl. An inverse association was identified between living at a higher altitude and the proportion of cardiometabolic risk in the Peruvian adult population. However, at least 8 out of 10 people were identified as at risk in all categories of altitude.

## 1. Introduction

The World Health Organization (WHO) considers obesity a global public health problem due to its constant increase and its impacts on mortality and the development of diseases, mainly metabolic and cardiovascular disorders [1]. In 2016, it was estimated that a quarter of the adult population of Latin America and the Caribbean (LAC) had obesity. Similarly, in the period from 2000 to 2016, significant increases in this condition were recorded in the LAC region and its subregions, ranging between 7.2 and 9.5% [2]. In Peru, in 2021, the prevalence of obesity was estimated at 25.8% in the population aged 15 years and over [3], having been preceded by a sustained increase in this condition in recent decades [4]. Due to the impacts of obesity on population morbidity and mortality, its monitoring as a cardiometabolic risk factor, as well as the evaluation of its main determining factors, are essential actions for protecting public health.

Cardiometabolic risk factors may differ according to geographical characteristics such as altitude. In Peru, an inverse association between altitude and body mass index (BMI) and abdominal obesity was reported using data from the years 2009 to 2013 [5,6]. Likewise, another study that used information from the Demographic and Family Health Surveys for the 2014–2020 period in Peru determined a similar association between altitude and predicted 10-year cardiovascular risk in the same population, measured from the version without laboratory parameters of a WHO tool that involves the measurement of body mass index (BMI) [7,8]. The problem with using BMI as a measure to predict cardiovascular risk is that it incorporates weight, which does not distinguish between lean and fat mass, thereby inadequately approximating body fat distribution [9,10,11]. In addition, the BMI is influenced by the height of the subjects, leading to those of shorter height having a higher BMI, although they may have less body fat [12]. On the other hand, although waist circumference (WC) has been shown to be a better measurement of adiposity and predictor of cardiometabolic risk than BMI in different populations, its use may be inappropriate in the Peruvian population in which there is great variability in height [13,14]. This is relevant because the anthropometric measurements and the calculated cardiometabolic risk can vary in high-altitude populations, and, in Peru, 18% of the population lives more than 2500 m above sea level (masl) [15].

Different studies have evaluated anthropometric indices in order to identify the best predictor of cardiometabolic risk. The weight–height ratio (WHtR) has shown better predictive power of cardiometabolic risk in different populations, including Peru, than other indices such as BMI or WC [16,17,18]. Likewise, changes in body composition patterns have been reported in different populations due to the COVID-19 pandemic [19]. Therefore, it is important to take into account the measurement of WHtR in the Peruvian population to identify the population at cardiometabolic risk with a higher level of sensitivity.

Knowing how geographic variables affect the distribution of individuals with cardiometabolic risk is important for defining preventive strategies for the appearance of diseases such as hypertension and diabetes. The objective of the present study was to evaluate the association between altitude and cardiometabolic risk calculated with the WHtR, using data from the Peruvian adult population reported by the Demographic and Family Health Survey (ENDES) 2021.

## 2. Materials and Methods

### 2.1. Data Source and Data Description

This cross-sectional study was developed with data from the National Demographic and Family Health Survey (ENDES) 2021 [3]. This is a population-based survey carried out between January and December 2021 by the National Institute of Statistics and Informatics (INEI), which applies three questionnaires: one addressed to the household and its members through a competent informant (household questionnaire), a second questionnaire aimed at all women aged 12 to 49 years (individual women’s questionnaire), and a third questionnaire applied to a chosen person aged 15 years and over (health questionnaire) [3]. The main objective of the health questionnaire is to provide information on non-communicable diseases, communicable diseases, and cancer prevention and control in people 15 years of age and older. Likewise, it collects information on access to diagnostic services and treatments for eye, oral, and mental health in girls and boys under 12 years of age. The target population is private households and their members, including one person 15 years of age or older for each private household as well as all girls and boys under 12 years of age [3]. The sampling framework is made up of the statistical and cartographic information from the National Census XII of Population and VII of Housing of the year 2017 (CPV 2017) and the cartographic material updated for this purpose in the process carried out for the execution of ENDES 2021 [3].

### 2.2. Sampling and Data Collection

The ENDES 2021 sample is characterized by being two-stage, probabilistic, balanced, independent, and stratified at the departmental level into urban and rural areas [20]. The sampling units in the urban areas are the conglomerate and private dwellings, and in the rural areas, they are the rural census area and private dwellings. The research unit of the health questionnaire is made up of people of the ages of interest who are regular residents of private homes in urban and rural areas of the country who spent the night before the survey in the home selected [3]. The collection of coverage information in the selected dwellings is carried out using a mobile device (tablet). The method used is a direct interview conducted by duly trained personnel to collect this information. More details about the sampling process, the design, and the contents of ENDES 2021 can be found in the annual report and technical sheet [3,20].

**Figure 1:**
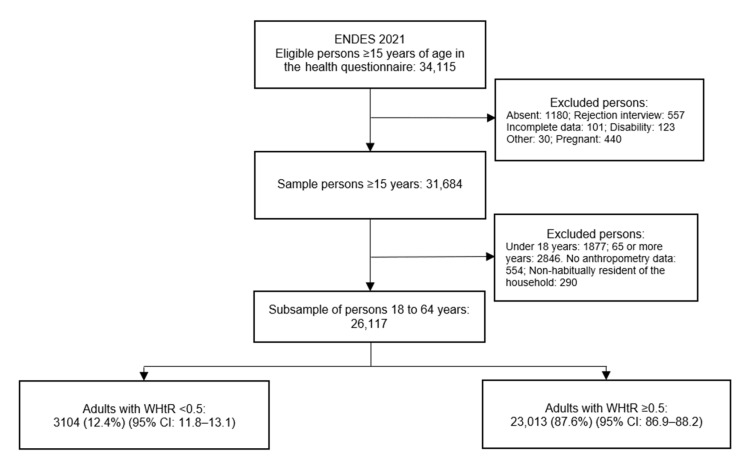
Flow chart of the selection of adults included in the study.

The final sample for the analysis was made up of 26,117 adult subjects (18 to 64 years) who were habitual residents of their households, after removing incomplete data in the variables of interest. The flow chart of the study is presented in Figure 1.

### 2.3. Dependent Variable

In this study, the dependent variable was cardiometabolic risk measured according to the WHtR in adults aged 18 to 64 years, which was dichotomized into “Yes = 1” if the WHtR was ≥0.5 and “No = 0” if the WHtR was <0.5. The WHtR was calculated by dividing the abdominal perimeter (cm) by the height (cm). The WHtR cut-off point for risk estimation was 0.5 or more, which has been recommended as a cut-off point in different sexes and ethnic groups and can be applied to both children and adults [16,21].

### 2.4. Exposure Variable

The exposure variable was defined as altitude measured in masl using the global positioning system (GPS) installed in a tablet and taking as a reference point one meter away from the main door of the household of the surveyed individual. More details on the process of taking and registering the GPS point of households and clusters can be found in the Interviewer’s Manual of ENDES 2021 [22]. Altitude was categorized as: <1500; 1500 to 2499; 2500 to 3499; or 3500 or more masl based on the definition of altitude and associated physiological changes described by Barry and Pollard [23].

### 2.5. Covariates

The following covariables were selected as potential confounders of the association between altitude levels and cardiometabolic risk, based on relevance and availability. Covariates included sex (men, women), age group (18–29, 30–39, 40–49, 50–64), educational level (up to primary, secondary, higher), wealth index (poorest, poorer, middle, richer, richest), and area of residence (urban, rural).

### 2.6. Statistical Analysis

The data analysis included a descriptive and inferential analysis that included the characteristics of complex sampling and the sample weights of ENDES 2021. Descriptive analysis was used to determine the frequency distribution of the study variables. Chi-square tests were performed to determine the differences between the proportions of the variables included in the study. Poisson log generalized linear regression models (bivariate and multivariate) were fitted to evaluate the association between altitude and WHtR, reporting prevalence ratios (PR) and 95% confidence intervals (CIs) as measures of association. In addition, we used Stata’s testparm post-estimation command to assess the global effects of sex and age as potential modifiers of the association between altitude and WHtR.

The level of statistical significance was 5%. All statistical analyses were performed in Stata 17 (StataCorp, College Station, TX, USA), and the svy command with the subpop option was used to estimate the study subpopulation (18 to 64 years).

### 2.7. Ethical Considerations

Ethical review and approval were waived for this study since all the data from the ENDES are publicly accessible. All the subjects agreed to complete the survey and provided consent. The datasets used in this work were released on the INEI website: http://iinei.inei.gob.pe/microdatos/ (accessed on 1 July 2022).

## 3. Results

A total of 26,117 participants were included. The majority were women (51.2%) from the age group of 18 to 29 years (31.2%) who had secondary education level (46%) and medium well-being index (21.3%) and lived in an urban area (81.6%). The mean WHtR in the population was 0.59 (standard deviation [SD]: 0.08) (see Table 1).

The largest proportion of the population lived at an altitude of less than 1500 masl (73.5%), followed by 2500 to 3499 masl (12.7%) (See Table 1). The highest mean WHtR was identified in the population residing at an altitude below 1500 masl (0.59; SD; 0.07), while the lowest mean was identified in the population living at more than 3500 masl (0.57; SD: 0.1). All study covariates showed an association with altitude, except for the variable of age group (See Table 2).

According to the WHtR, 87.6% (95% CI: 86.9–88.2) of the total population was classified as at risk, and all covariates were significantly associated with cardiometabolic risk calculated by the WHtR (See Table 3).

In the regression analysis adjusted for sex, age, education level, well-being index, and area of residence and taking as reference persons residing at less than 1500 masl, living at altitudes between 2500 and 3499 masl (aPR: 0.98; 95% CI: 0.96–1.00) or over 3500 m (aPR: 0.95; 95% CI: 0.93–0.97) was associated with a lower proportion of the population at cardiometabolic risk (see Table 4). The interaction term for sex and altitude was statistically significant (Stata testparm command, *p* < 0.001); therefore, we present the analysis stratified by sex (see Table 4). In men, the categories 2500–3499 and 3500 or more masl remained significantly associated, while in women, only the 3500 or more masl category remained significantly associated (see Table 4). The interaction term for age and altitude was not statistically significant (Stata testparm command, *p* = 0.222).

## 4. Discussion

The objective of this study was to evaluate the association between altitude and cardiometabolic risk, calculated using the WHtR in the Peruvian adult population. After adjustment for potential confounders, it was identified that residents at altitudes of 2500 to 3499 masl and 3500 masl and above had 2% and 5% lower prevalence of cardiometabolic risk, respectively, compared with residents at less than 2500 or 1500 masl.

The association identified has been previously reported in a study that used data from the National Household Survey in Peru for the years 2012–2013 [5]. In that study, an inverse association was identified between altitude and abdominal obesity, with prevalences of abdominal obesity calculated by the WHtR of 86.1%, 80.7% and 77.9%, although with a different categorization of altitude (<1500 masl; 1500 to 2999 masl; ≥3000 masl) and without adequately supporting said categorization. However, we identified a higher prevalence of cardiometabolic risk (88.5%) for the only category similar to that in our analysis (<1500 masl), which supports the hypothesis of an increment in cardiometabolic risk in the population of low- and middle-income countries [24]. In the literature, various mechanisms have been postulated to explain this association, ranging from exposure to chronic hypoxic states, increased basal metabolic rates, lack of appetite, dehydration, and intestinal malabsorption [25]. However, the exact mechanisms of the association under study are not yet conclusively known. With regard to the association stratified by sex, the associated categories of altitude only remained significant in men, while in women only the 3500 or more category was significantly associated with cardiometabolic risk. A similar finding was reported in a previous study that evaluated the association between altitude and obesity [6]. Although, these findings may suggest a moderating effect, these results should be considered exploratory until the possible mechanisms can be adequately explained.

Although the present results show a significantly lower proportion of cardiometabolic risk in the population living at more than 3500 masl, this was above 82% in all the altitude categories evaluated. Although this percentage is expected in lower-altitude areas, due to the high urbanization rates in cities mainly on the Peruvian coast, it is striking how the risk is similar even at high altitudes at which rurality prevails. This phenomenon was previously reported in a study that used data from 200 countries and evaluated the trajectories of urban–rural differences in BMI in the period 1985 to 2017 and observed that the growth rate of the BMI is similar or higher in rural to that in urban areas, mainly in low- and middle-income regions [26]. Although this can be explained by changes in eating habits, poor food quality, and low physical activity in rural areas, it should be taken into account that in high-altitude cities in Peru, as in other countries with few or limited resources, rural areas also present other limitations that may be related to higher cardiometabolic risk and consequent morbidities, such as poor access to health services, lack of specialized personnel, long transport distances to health centers, lack of public insurance, and higher out-of-pocket spending [27,28]. In this sense, health policies that seek to mitigate the effect of cardiometabolic risk factors at the structural and individual levels should be strengthened, with emphasis on rural areas, which remain neglected. Thus, measures such as training in primary prevention for medical and non-medical health personnel, as well as the strengthening of telemedicine, could be very useful.

On the other hand, the lower prevalence of cardiometabolic risk in the higher-altitude areas in Peru reported by ours and other studies [7,29] may be due to the high figures of poverty and, consequently, of malnutrition in comparison with the areas of lower altitude [30]. Paradoxically, this could generate a false idea of a better cardiometabolic profile in the population living at higher altitudes when measured according to the distribution of body adiposity, with the lower measurements actually being due to a higher frequency of malnutrition, which could be independently associated with increased cardiometabolic risk [31]. 

The use of the WHtR is relevant in Peru considering the great variability in the height of the population [13]. The use of the WHtR accounts for abdominal adiposity while also taking into account the person’s height, thereby making this an ideal measurement for estimating cardiometabolic risk in our country. Likewise, this index also offers greater precision for the detection of cardiometabolic risk [16,18], as well as the presentation of diseases such as hypertension and type 2 diabetes [17]. Similarly, the use of the BMI and WC may underestimate cardiometabolic risk compared with the WHtR in different populations [17,32], classifying some people at risk as healthy. Therefore, the implementation of the WHtR should be extended as an anthropometric index in Peru for clinical and research purposes, being also cheaper and easier to carry out than the BMI.

One the main limitations of this study is the transversal design, which lacks the criterion of temporality necessary for determining causality between exposure (altitude) and outcome (cardiometabolic risk). Likewise, there is a risk of residual confounding due to the impossibility of measuring the confounding variables such as migratory status or the time from migration between the altitudinal levels considered in the present study. Errors may have been made in the measurement of the anthropometric parameters necessary for the calculation of the WHtR during the survey, such as the WC and height, thereby introducing a bias of incorrect outcome classification. However, the present study used data from the ENDES, which has national representativity and also uses the methodological framework of the Demographic and Health Surveys (DHS). The DHS are surveys that have been carried out since 1984 and are applied in more than 90 countries around the world. Interviewers and anthropometrists are properly trained to be able to collect information with the greatest possible precision. Finally, our study reports relevant results on the association between altitude and cardiometabolic risk measured with the WHtR in Peruvian adults, which in previous studies has been shown to be a better predictor of cardiometabolic risk than the BMI or WC in the Peruvian population.

## 5. Conclusions

In conclusion, an inverse association was identified between living at a higher altitude and the level of cardiometabolic risk in the Peruvian adult population. However, the prevalence of cardiometabolic risk in the different altitude categories evaluated remains above 82%, which represents a large proportion of the population at risk every altitude. Taking this into account, in Peru, it is necessary to strengthen public health strategies in the populations that live at low altitudes; moreover, a potential increase in cardiometabolic risk should be anticipated in the population living at higher altitudes. Future studies should confirm this association using longitudinal designs as well as clarifying the doubts about the underlying mechanisms.

## Figures and Tables

**Table 1:** Characteristics of the participants included in the study (*n* = 26,117).

Characteristics	*n*	% *
Sex		
Men	11,114	48.8
Women	15,003	51.2
Age groups (years)		
18–29	8303	31.2
30–39	8903	24.9
40–49	4665	21.1
50–64	4246	22.8
Education level		
Up to primary	5730	17.6
Secondary	12,054	46.0
Higher	8333	36.4
Wealth Index		
Poorest	8159	18.2
Poorer	6756	20.8
Middle	4900	21.3
Richer	3765	20.7
Richest	2537	19.1
Area of residence		
Urban	17,180	81.6
Rural	8937	18.4
Altitude of residence (in masl)		
<1500	16,125	73.5
1500–2499	2263	6.7
2500–3499	4540	12.7
3500 or more	3189	7.2
Weight–height ratio		
Mean (SD)		0.59 (0.08)

* Estimates include the weights and ENDES 2021 sample specifications. Values are % unless otherwise indicated. SD: standard deviation; masl: meters above sea level.

**Table 2:** Characteristics of the participants according to the altitude of residence.

	Altitude				
	<1500 (*n* = 16,125)	1500–2499 (*n* = 2263)	2500–3499 (*n* = 4540)	3500 or More (*n* = 3189)	
Characteristics	% (95% CI)	% (95% CI)	% (95% CI)	% (95% CI)	*p* Value *
Weight–Height Ratio, mean (SD)	0.594 (0.07)	0.581 (0.09)	0.579 (0.09)	0.575 (0.1)	<0.001
Waist to Height Risk					
No	11.5 (10.7–12.3)	14.0 (12.0–16.1)	14.5 (13.0–16.0)	17.2 (15.4–19.1)	<0.001
Yes	88.5 (87.7–89.3)	86.0 (83.9–88.0)	85.5 (84.0–87.0)	82.8 (80.9–84.6)	
Sex					
Men	49.0 (47.9–50.2)	51.3 (48.5–54.1)	47.6 (45.7–49.4)	46.3 (44.0–48.7)	0.040
Women	51.0 (49.8–52.1)	48.7 (45.9–51.5)	52.4 (50.6–54.3)	53.7 (51.3–56.0)	
Age groups (years)					
18–29	31.1 (30.0–32.2)	30.8 (28.1–33.6)	32.4 (30.4–34.4)	30.2 (28.0–32.5)	0.424
30–39	25.1 (24.2–26.1)	24.7 (22.6–27.0)	24.5 (23.0–26.1)	23.8 (22.2–25.6)	
40–49	21.2 (20.2–22.2)	20.7 (18.4–23.2)	21.1 (19.6–22.7)	20.3 (18.2–22.5)	
50–64	22.5 (21.5–23.6)	23.8 (21.1–26.6)	22.0 (20.1–24.0)	25.7 (23.7–27.8)	
Educational level					
Up to primary	13.6 (12.8–14.4)	26.7 (23.5–30.2)	27.0 (24.8–29.3)	33.2 (30.3–36.1)	<0.001
Secondary	48.0 (46.7–49.3)	37.9 (34.8–41.0)	40.9 (38.7–43.1)	42.7 (40.1–45.3)	
Higher	38.4 (37.1–39.7)	35.4 (32.1–38.9)	32.2 (30.0–34.4)	24.1 (21.8–26.7)	
Wealth Index					
Poorest	10.0 (9.3–10.8)	35.6 (30.7–40.8)	37.3 (34.2–40.4)	51.7 (47.7–55.8)	<0.001
Poorer	19.8 (18.8–20.9)	18.3 (15.6–21.3)	25.2 (23.1–27.4)	25.1 (22.1–28.5)	
Middle	23.1 (22.0–24.3)	17.4 (14.4–20.9)	17.8 (16.1–19.6)	13.0 (10.8–15.6)	
Richer	23.9 (22.8–25.1)	13.9 (11.5–16.7)	12.6 (10.9–14.6)	7.5 (6.1–9.2)	
Richest	23.1 (21.8–24.4)	14.9 (12.4–17.8)	7.2 (6.1–8.5)	2.6 (1.7–3.9)	
Area of residence					
Urban	90.7 (89.7–91.5)	57.4 (51.6–63.0)	62.6 (58.8–66.2)	44.3 (40.2–48.4)	<0.001
Rural	9.3 (8.5–10.3)	42.6 (37.0–48.4)	37.4 (33.8–41.2)	55.7 (51.6–59.8)	

Estimates include the weights and ENDES 2021 sample specifications. * The *p*-value was calculated using the Rao–Scott Chi-squared test or F test. Values are % unless otherwise indicated. CI: confidence interval; SD: standard deviation.

**Table 3:** Characteristics of the participants according to weight–height ratio.

	Waist to Height Risk		
	No (*n* = 3104)	Yes (*n* = 23,013)	
Characteristics	% (95% CI)	% (95% CI)	*p* Value *
Overall	12.4 (11.8–13.1)	87.6 (86.9–88.2)	
Sex			
Men	66.4 (64.0–68.7)	46.3 (45.3–47.3)	<0.001
Women	33.6 (31.3–36.0)	53.7 (52.7–54.7)	
Age groups (years)			
18–29	69.8 (67.4–72.0)	25.7 (24.9–26.6)	<0.001
30–39	16.2 (14.6–18.0)	26.2 (25.4–27.0)	
40–49	6.8 (5.7–8.1)	23.1 (22.3–24.0)	
50–64	7.2 (5.8–8.8)	25.0 (24.1–25.9)	
Education level			
Up to primary	12.3 (10.8–13.9)	18.3 (17.6–19.0)	<0.001
Secondary	51.2 (48.5–53.8)	45.3 (44.2–46.4)	
Higher	36.6 (34.0–39.2)	36.4 (35.3–37.4)	
Wealth Index			
Poorest	24.5 (22.6–26.4)	17.3 (16.6–17.9)	<0.001
Poorer	21.1 (19.1–23.4)	20.7 (19.9–21.6)	
Middle	18.7 (16.6–21.1)	21.7 (20.8–22.6)	
Richer	18.0 (15.9–20.3)	21.0 (20.1–22.0)	
Richest	17.6 (15.4–20.2)	19.3 (18.3–20.3)	
Area of residence			
Urban	75.8 (73.9–77.6)	82.4 (81.8–83.0)	<0.001
Rural	24.2 (22.4–26.1)	17.6 (17.0–18.2)	

Estimates include the weights and ENDES 2021 sample specifications. * The *p*-value was calculated using the Rao–Scott Chi-squared test. CI: confidence interval.

**Table 4:** Association between altitude and weight–height ratio.

Characteristics	Crude Model		Adjusted Model	
	PR (95% CI)	*p* Value	aPR (95% CI)	*p* Value
Overall				
Altitude (masl) *				
<1500	Reference		Reference	
1500–2499	0.97 (0.95–1.00)	0.280	0.99 (0.96–1.01)	0.341
2500–3499	0.97 (0.95–0.99)	0.001	0.98 (0.96–1.00)	0.024
3500 or more	0.94 (0.91–0.96)	<0.001	0.95 (0.93–0.97)	<0.001
Men				
Altitude (masl) **				
<1500	Reference		Reference	
1500–2499	0.95 (0.91–0.99)	0.017	0.97 (0.93–1.01)	0.150
2500–3499	0.90 (0.87–0.94)	<0.001	0.94 (0.91–0.97)	0.001
3500 or more	0.90 (0.86–0.94)	<0.001	0.94 (0.90–0.99)	0.011
Women				
Altitude (masl) **				
<1500	Reference		Reference	
1500–2499	1.00 (0.98–1.02)	0.946	1.00 (0.98–1.03)	0.680
2500–3499	1.02 (1.00–1.03)	0.058	1.01 (1.00–1.03)	0.157
3500 or more	0.96 (0.94–0.98)	0.001	0.95 (0.93–0.97)	<0.001

Estimates include the weights and ENDES 2021 sample specifications. * Adjusted for sex, age, educational level, wealth index, and area of residence. ** Adjusted for age, educational level, wealth index, and area of residence. masl: meters above sea level; PR: prevalence ratio; aPR: adjusted prevalence ratio; CI: confidence interval.

## Data Availability

Not applicable.
